# Protein dysregulation during *Leishmania infantum* infection in anti-TNF immunosuppressed mice revealed through quantitative proteomics analysis of extracellular vesicles

**DOI:** 10.3389/fimmu.2025.1634080

**Published:** 2025-07-30

**Authors:** Lorena Bernardo, Ana Montero-Calle, Jose Carlos Solana, Marina Lozano-Rendal, Ana Torres, Carmen Sánchez, Rodrigo Barderas, Javier Moreno, Eugenia Carrillo

**Affiliations:** ^1^ WHO Collaborating Centre for Leishmaniasis, Spanish National Center for Microbiology, Instituto de Salud Carlos III, Madrid, Spain; ^2^ Centro de Investigación Biomédica en Red de Enfermedades Infecciosas (CIBERINFEC), Instituto de Salud Carlos III, Madrid, Spain; ^3^ Chronic Disease Program (UFIEC), Instituto de Salud Carlos III, Madrid, Spain; ^4^ Centro de Investigación Biomédica en Red de Fragilidad y Envejecimiento Saludable (CIBERFES), Instituto de Salud Carlos III, Madrid, Spain

**Keywords:** extracellular vesicles, visceral leishmaniasis, immunosuppression, TNF antagonist, antimonials, quantitative proteomics, LFQ proteomics analyses, biomarkers

## Abstract

**Introduction:**

Visceral leishmaniasis (VL) occurs more frequently in immunosuppressed individuals, especially those undergoing immunosuppressive drug therapy for an autoimmune disease. In those receiving TNF antagonist therapy (anti-TNF), the course of VL is more severe and the response to traditional leishmanicidal treatments, such as antimonials (Sb), is often reduced. This effect of anti-TNF treatment is observed in our immunosuppressed-mouse model of VL. In this model, we compared anti-TNF immunosuppression with no immunosuppression before and after VL treatment with Sb.

**Methods:**

Serum-derived extracellular vesicles (EVs) were analyzed through label-free quantitative proteomics to identify proteins involved in both VL severity and the impact of anti-TNF immunosuppression on treatment outcome.

**Results:**

In total, 223 dysregulated proteins were found in the pre-treatment groups, the majority of which, such as vitronectin, haemopexin or caveolin-1, were downregulated in the anti-TNF samples. In contrast, 173 proteins were identified in the Sb-treatment groups, most of which were found enriched in the anti-TNF plus treatment samples (anti-TNF+Sb) including fibronectin, transferrin, vitronectin and dipeptidyl peptidase-4. These differentially-expressed proteins were associated with pathways related to the immune system, liver regeneration, and ion transport.

**Conclusion:**

Our findings have useful implications for the clinical management of VL patients under anti-TNF immunosuppression.

## 1 Introduction

Leishmaniasis is a neglected vector-borne tropical disease, whose most severe form –visceral leishmaniasis (VL)– is fatal if left untreated (1). Recently, an increasing number of cases of VL have been reported among individuals receiving immunosuppressive therapy to treat autoimmune diseases such as psoriasis or rheumatoid arthritis (2). Anti-TNF therapies have improved the quality of life of these patients by reducing the inflammatory effects of the TNF cytokine. However, by reducing the protective capacity of the immune system, the patient becomes more susceptible to opportunistic pathogens such as those causing VL. This occurs because TNF plays an important role in the activation and differentiation of immune cells such as macrophages (3, 4). In addition, immunosuppression, particularly with anti-TNF therapies, is responsible for approximately 50% of VL cases among immunocompromised individuals, which compromise the efficacy of VL treatments and, consequently, increase the risk of VL relapse (5, 6). Thus the challenges faced by these immunosuppressed VL patients are exacerbated, especially given the currently limited availability of antileishmania chemotherapeutic agents.

Considering that immunosuppression is the main individual risk factor for a person to develop VL, there is a real need to understand the mechanisms driving the increased disease severity and reduced efficacy of leishmanicidal treatments observed in these immunocompromised patients. Few studies have addressed this issue as most of the pertinent literature has focused on describing the severe symptoms and complications experienced by these patients. We early reported in a mouse model of VL, that the administration of anti-TNF antibodies modulates the natural course of *Leishmania infantum* infection, drastically increasing parasite load in the liver and suppressing the Th1-protective effect of CD4+ and CD8+ T cells when compared to what happens in immunocompetent mice (7). Factors found to contribute to parasite persistence and disease severity were a lack of the production of pro-inflammatory cytokines such as IFN-γ, along with an increase in IL-10-producing regulatory T cells (Treg) and in the cell exhaustion marker PD-1 (8–10). We also described that the lowest response to leishmanicidal treatment with pentavalent antimonials (Sb) in anti-TNF mice, with respect to the immunocompetent group, was mainly the consequence of reduced activation of the immune system’s defence capacity (11, 12). Under this specific immunosuppression, Sb therapy failed to promote the recruitment of dendritic cell populations, and, instead, increased the frequencies of IL-10 and PD-1-producing B cells. This diminished antigen presentation and the reduced activation of T lymphocytes could explain the greater risk of relapse observed in clinical cases of VL receiving anti-TNF immunosuppression therapy.

As these cell mechanisms affected by anti-TNF immunosuppression become clearer, more research is needed to improve the clinical management of these patients in endemic areas of leishmaniasis. Clinically useful biomarkers are urgently needed to monitor the efficacy of leishmanicidal treatments in anti-TNF immunosuppressed patients (13). In recent years, extracellular vesicles (EVs) have revolutionised the field of biomarker research due to their roles in host-parasite communication, the immune response and drug resistance, among others (14–17). EVs are small particles enclosed in a lipid bilayer membrane (18) that contain a wide range of biomolecules, such as proteins, that may serve as biomarkers (19, 20), particularly those derived from readily-available samples like blood plasma or serum.

To gain insight into the underlying mechanisms of VL and its treatment, the primary aim of this study was to compare the protein contents of EVs using a proteomic approach, in serum samples from *Leishmania*-infected non-immunosuppressed and anti-TNF- immunosuppressed mice before and after Sb treatment. Subsequently, the most prominent proteins were evaluated by ELISA in serum to assess whether the candidate proteins identified in EVs could also be detectable and relevant in an easily accessible in clinical matrix for clinical purposes. Our ultimate goal was to identify proteins that could be associated with the prognosis and treatment response to VL under anti-TNF immunosuppression.

## 2 Materials and methods

### 2.1 Sample collection

**Figure 1:**
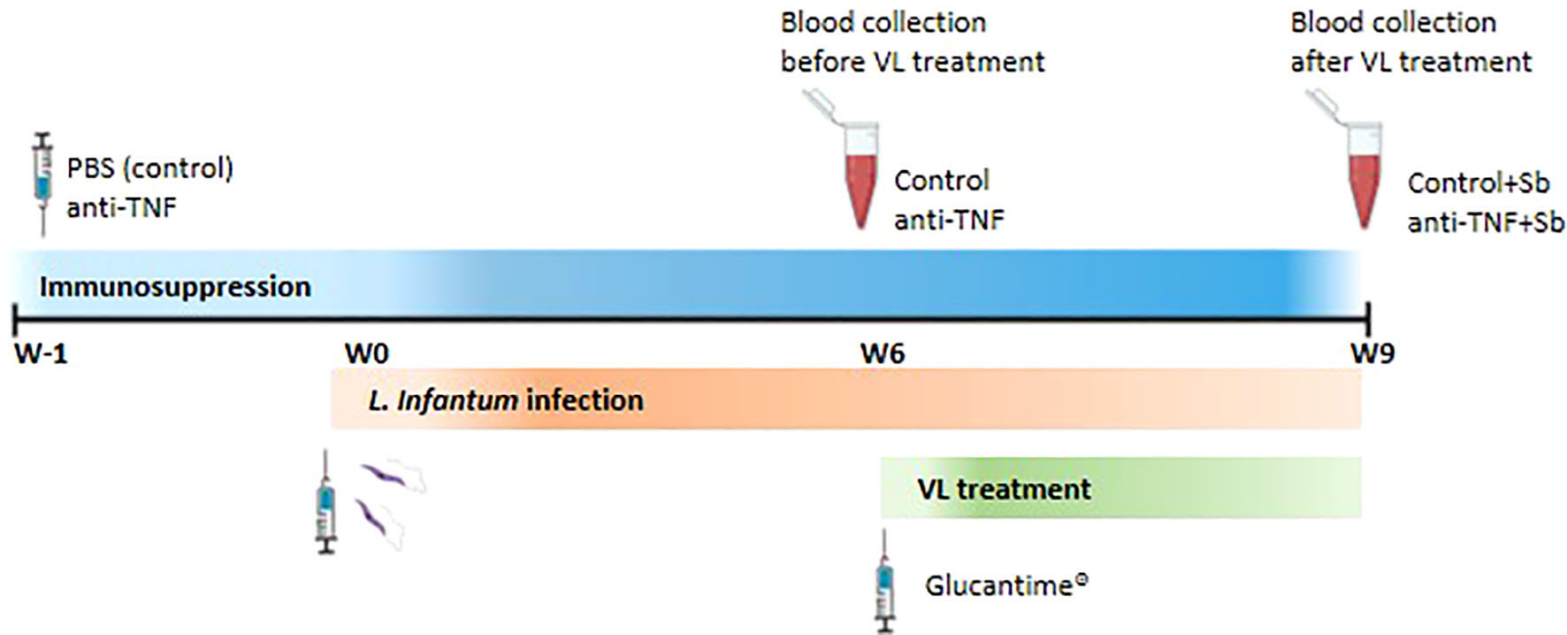
Schematic representation of the experimental design. BALB/c mice were randomly divided into two groups and received intraperitoneal (i.p.) administrations of either PBS (control group) or anti-TNF at 20 mg/kg twice weekly. These regimens were maintained throughout the duration of the experiment. One week after the initiation of immunosuppressive treatment (week 0), mice were intravenously infected with *L. infantum* promastigotes. At six weeks post-infection (W6), blood samples were collected for serum isolation prior to VL treatment. At this point, two additional groups of mice –previously immunosuppressed and infected- began a 21-day course of Glucantime treatment (20 mg/kg 7day, i.p.), forming the control+Sb and anti-TNF+Sb groups. A second blood collection was performed at the end of the treatment period (W9).

Serum samples were obtained from a VL model in anti-TNF immunosuppressed BALB/c mice intravenously infected with 1 x10^7^ *L. infantum* promastigotes (12). Mice were assigned to the groups (n=6 each): control, PBS 1X (Gibco, USA); anti-TNF, 20 mg/kg (Leinco Technologies, USA); control+Sb, PBS + Glucantime^®^ (Sanofi, France) and anti-TNF+Sb, anti-TNF + Glucantime^®^ (**Figure 1**). The control and anti-TNF groups were administered PBS or anti-TNF twice per week and blood samples collected after six weeks of infection. The control+Sb and anti-TNF+Sb groups were administered PBS or anti-TNF twice per week, and Sb was given in the last 21 days of the experiment prior to blood collection after nine weeks of infection. Serum was isolated from blood samples (200 µL) collected from the submaxillary vein by centrifugation at 17,000 g for 15 min and kept at -20°C until use.

BALB/c mice used in this study were specific pathogen-free (SPF) and housed under controlled environmental conditions in individually ventilated cages. Food and water were provided ad libitum, both having been previously autoclaved to ensure sterility. All procedures were approved by the Committee on Ethics and Animal Welfare of the Instituto de Salud Carlos III (CBA 04_2018, PROEX 072/18) and animals handled according to Spanish legislation for the protection of animals used for scientific purposes (Royal Decree 53/203, law 32/2007).

### 2.2 Extracellular vesicle isolation

For EV isolation, a homogeneous 1 mL pool of serum was prepared for each study group. These samples were centrifuged at 300 g for 10 min and the supernatant diluted with an equal volume of filtered PBS to reduce viscosity. Next, the samples were centrifuged at 2,000 g for 30 min and at 12,000 g for another 30 min to pellet larger vesicles. EVs were than isolated from this final supernatant by size exclusion chromatography (SEC) followed by ultracentrifugation (UC) as previously described (21). Briefly, serum samples were first overlaid on the 70nm/qEV size exclusion column (Izon Science, New Zealand) and the flow-through was collected in 500 µL fractions. Next, fractions 7 to 9 were pooled and subjected to two ultracentrifugation steps at 100,000 g and 4°C for 2 h and 30 min, in a Beckman Coulter Optima XPN-100 ultracentrifuge with a swinging-bucket rotor (SW60Ti; Beckman Coulter Inc, USA). Finally, the pellet was resuspended in a final volume of 400 µL filtered PBS.

### 2.3 EV sample characterization

The protein contents of the serum-derived EVs were measured with the BCA Protein Assay Kit (Thermo Fisher Scientific, USA) following the manufacturer’s instructions. Briefly, 10-µL aliquots were used in the reaction mixture followed by incubation for 30 min at 37°C and absorbance measurement at 562 nm. Protein concentration was calculated based on a standard curve prepared with bovine serum albumin (BSA) and fitted using a four-parameter logistic model.

Particle size and concentration measurements of the isolated EVs were made in each sample using a NanoSight NS300 instrument (Malvern, Worcestershire, UK) and analysed using NTA 3.2 software. Samples were diluted 1:50 in filtered PBS and run with default settings. Mean particle size and concentration were calculated based on three independent records (60-s each) obtained for each sample.

Finally, EV samples were examined by transmission electron microscopy (TEM) through negative staining on glow-discharged carbon-coated copper grids. Preparations were fixed in 2% paraformaldehyde for 5 min and then washed two times with MiliQ water and negatively stained with 2% aqueous uranyl acetate for 1 min. Particles were visualized using a FEI Tecnai 12 electron microscope equipped with a LaB6 filament operating at 120 kV. Images were captured using a FEI Ceta digital camera at a nominal magnification of 28,000x (Microscopy Service of the National Centre for Microbiology, Instituto de Salud Carlos III).

### 2.4 Label free quantification proteomics

To assess protein dysregulation associated with anti-TNF immunosuppression and Sb treatment, we used a label free quantification (LFQ) proteomics approach. To this end, 2.8 µg of each EV sample were resuspended in lysis buffer (RIPA, Sigma-Aldrich, USA), made up to a final volume of 150 µL, and incubated 5 min on ice and 5 min at 95°C (x5) to ensure complete lysis of the EVs. The protein extracts were then reduced in 1:10 diluted 100 mM tris (2-carboxyethyl) phosphine (TCEP, Sigma-Aldrich) for 45 min at 37°C and 1,000 rpm, and alkylated with 1:10 diluted 400 mM chloroacetamide (Sigma-Aldrich) for 30 min at room temperature (RT) and 1,000 rpm in the dark. For protein anchoring, the reduced extracts were incubated with 100 µL of SeraMag magnetic beads mix (2.5 µL of hydrophilic beads-2.5 µL of hydrophobic beads per sample, Cytiva, UK) and 200 μL of 100% acetonitrile (ACN) for 35 min at RT and 1,000 rpm. Next, the magnetic beads were washed twice with ethanol 70% and once with ACN 100%, and, finally, beads were incubated overnight at 37°C with 0.2 μg of porcine trypsin (Thermo Fisher Scientific) in 100 μL of 50 mM ammonium bicarbonate, pH 8.0. To recover peptides, the samples were sonicated twice and supernatants containing the digested proteins collected, dried under vacuum, and stored at -80°C until analysis in an Orbitrap Exploris 480 mass spectrometer equipped with a FAIMS pro Duo interface (22).

### 2.5 LC-MS/MS analysis

Peptide samples were resuspended in 11 μL of 0.1% formic acid (FA) H_2_O and 2 μL of each sample (800 ng) were injected four times using the Vanquish Neo UHPLC System (Thermo Fisher Scientific). For liquid chromatography (LC), samples were loaded onto a precolumn PepMap 100 C18 3 µm, 75 µm × 2 cm Nanoviper Trap 1200BA (Thermo Fisher Scientific) and eluted in an Easy-Spray PepMap RSLC C18 2 µm, 75 µm × 50 cm (Thermo Fisher Scientific) heated to 50°C. The mobile phase flow rate was 300 nL/min using 0.1% FA H_2_O (buffer A) and 0.1% FA in 80% ACN (buffer B). The 2-h elution gradient was: 2% buffer B for 5 min, 2-20% buffer B for 100 min, 20-42% buffer B for 10 min, 42–95% buffer B for 1 min, and 95% buffer B for 10 min.

For tandem mass spectrometry (MS/MS) analysis, 2300 V of liquid junction voltage and 280°C capillary temperature were used for ionization. The full scan method employed a m/z 350–1400 mass selection, an Orbitrap resolution of 60,000 (at m/z 200), an automatic gain control (AGC) value of 300%, and a maximum injection time (IT) of 25 ms. For MS2, the 12 most intense precursor ions were selected for fragmentation with a normalized collision energy of 32. MS2 scans were acquired with a 100 m/z first mass, an AGC target of 200%, a resolution of 15,000 (at m/z 200), an intensity threshold of 5×10^4^, an isolation window of 1.3 m/z, and a maximum IT of 22 ms. Charge state screening was enabled to reject unassigned, singly charged, and greater than or equal to seven protonated ions. A dynamic exclusion time of 30 s was used to discriminate against previously selected ions. For FAIMS, a gas flow of 4 L/min and CVs = -45 V and -60 V were used.

### 2.6 MS data analysis and Statistical analysis

MS data were analyzed with MaxQuant (version 2.1.3) using standardized workflows. Mass spectra *.raw files were searched against the Uniprot UP000000589_10090.fasta *Mus musculus* (mouse), accessed in October 2023 (17,114 protein entries) through standard procedures. Trypsin/P was specified as cleavage enzyme, and precursor and reporter mass tolerances were set to 4.5 ppm and 0.003 Da, respectively, allowing 2 missed cleavages. Carbamidomethylation of cysteines was set as a fixed modification, and methionine oxidation, N-terminal acetylation, and Ser, Thr, and Tyr phosphorylation were set as variable modifications. Label-free quantification and Fast LFQ were selected, with a LFQ minimum number of neighbours of 3, and a LFQ average number of neighbours of 6. Unique and razor peptides were considered for quantification. Minimal peptide length and maximal peptide mass were fixed to 7 amino acids and 4600 Da, respectively. Identified peptides were filtered by their precursor intensity fraction with a false discovery rate (FDR) threshold of 0.01. Proteins identified with at least one unique peptide and an ion score above 99% were considered for evaluation, whereas proteins identified as potential contaminants were excluded from the analysis. Protein sequence coverage was estimated as the percentage of matching amino acids from the identified peptides having a confidence level greater than or equal to 95% in the specific proteins divided by the total number of amino acids in the sequence.

Raw proteomics data obtained with the Orbitrap Exploris 480 mass spectrometer equipped with FAINS pro DUO interface were deposited to the ProteomeXchange Consortium via the PRIDE partner repository with the dataset identifier PXD060935.

Sample loading normalization was performed with R Studio (version 4.1.1) according to established protocols (https://github.com/pwilmart, accessed on 2 November 2022), using the “tidyverse”, “psych”, “gridExtra”, “scales”, and “ggplot2” packages (version 4.1.1). Finally, statistical analysis was performed using an empirical Bayes-moderated t-statistics method in R Studio (version 4.1.1) using the packages “limma”, “dplyr”, “tidyverse”, “ggplot2”, and “rstatix”, according to previously described procedures (22–24). Only proteins identified in at least 60% of samples analyzed in each comparison were considered for the analysis, and missing values imputed by random draws from a gaussian using the “imputeLCMD” R package.

### 2.7 Biological analysis

Functional annotations of the identified proteins in the EV preparations were obtained using the Database for Annotation, Visualization and Integrated Discovery (DAVID 2021) (25, 26). A gene ontology (GO) enrichment analysis was performed for cellular component and biological processes. Pathways with a *p*-value ≤ 0.05 and FDR ≤ 0.01 were taken into consideration. Results were created using Graphpad Prism software version 9.0 (GraphPad Software, USA), Functional Enrichment Analysis tool (FunRich) (27), and Flaski tool box (version 3.16.22) (28).

### 2.8 Enzyme-linked immunosorbent assay on serum

100 µL of both individual serum samples and standard curve solutions were added to pre-coated plates specific for each protein. The proteins assayed were Cav1 (serum dilution 1:2; EM0904, Fine Test), Dpp4 (1:100; orb391051, Biorbyt), Fn (1:500; EM0079, Fine Test), Hmgb-1 (1:10; orb1807923, Biorbyt), Hpx (1:100,000; EM2003, Fine Test), Tf (1:1000; EM1426, Fine Test) and Vtn (1:100; orb565383, Biorbyt). Dilutions of the serum samples were optimized according to the manufacturer’s instructions.

In brief, after an incubation of 90 min at 37°C, the plates were washed and biotin-conjugated detection antibody added for 60 min at 37°C. Next, streptavidin was added and the plates incubated for 30 to 45 min, depending on the specific protein. Following an additional wash, the plates were incubated with 3,3’,5,5’-tetramethylbenzidine (TMB) substrate solution and the reaction stopped with the stop solution provided in each ELISA kit. Absorbance was measured at 450 nm in a Multiskan FC microplate reader (Thermo Fisher Scientific, USA). Data analysis was performed using a four-parameter logistic curve implemented in GraphPad Prism version 9.0. Normally distributed data were analysed via ANOVA, followed by a Tukey’s *post hoc* test for multiple comparison. Significance was set up at p ≤ 0.05.

## 3 Results

### 3.1 Characterization and protein profiles of serum-derived EVs

**Figure 2:**
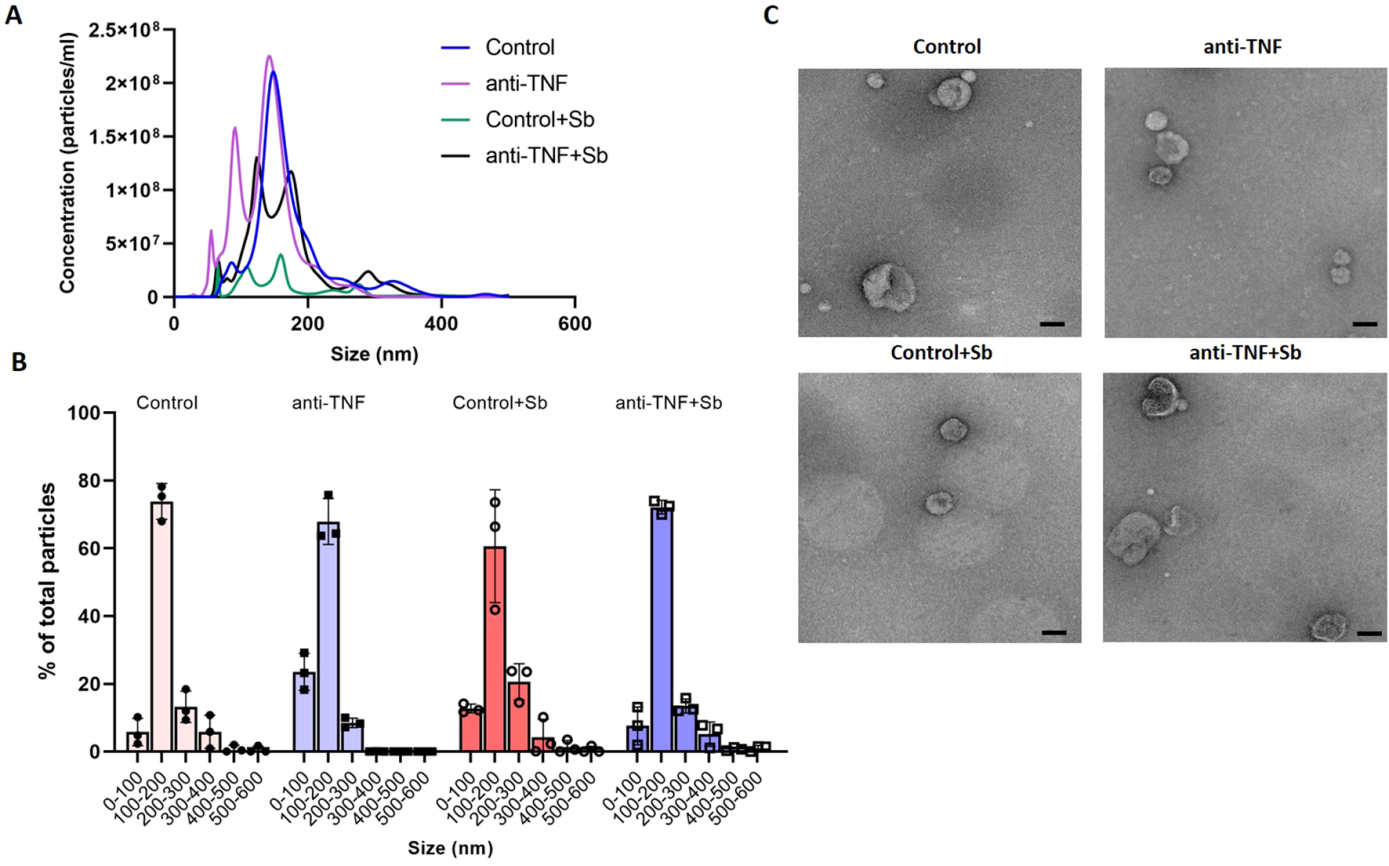
Characterization of EVs from control and anti-TNF samples before and after Sb treatment. **(A)** NTA was used to determine particle sizes and concentrations to identify the total number of isolated EVs. Although differences in concentrations were found, the distribution profile was similar across the groups. **(B)** Particle size distribution profile shown as the percentage of total particles detected within each sample. **(C)** EV morphology and size were determined by TEM. Scale bar set at 100 nm.

After purification of EVs by SEC and UC, samples were first characterized in terms of particle size and concentration by NTA, with a distribution profile comparable across the groups despite differences in concentrations (**Figure 2A**). In all groups, average EV size was in the range 100 to 200 nm, representing 73.80% (control), 67.91% (anti-TNF), 60.60% (control+Sb), and 72.13% (anti-TNF+Sb) of the total number of particles isolated (**Figure 2B**). Additionally, mean particle size was similar among groups, with no significant differences between them: 175.5 ± 1.9 nm (control), 172.9 ± 3.8 nm (control+Sb), and 173.3 ± 5.1 nm (anti-TNF+Sb). EVs recovered from the anti-TNF group showed the lowest mean size (137.3 ± 3 nm). In addition, NTA analysis confirmed an efficient isolation process with a particle concentration above 10^9^ in all extracts: 7.06 x 10^9^ ± 2.36 x 10^8^ (control), 8.29 x 10^9^ ± 4.55 x 10^8^ (anti-TNF), 1.34 x 10^9^ ± 1.34 x 10^8^ (control+Sb) and 5.92 x 10^9^ ± 2.96 x 10^8^ (anti-TNF+Sb) particles/mL. Next, the presence of EVs in all the preparations was confirmed by TEM (**Figure 2C**). In all images, it was possible to observe lipid bilayer-enveloped structures with a characteristic cup-shaped appearance, with maximum diameters in the range 100 to 200 nm.

**Figure 3:**
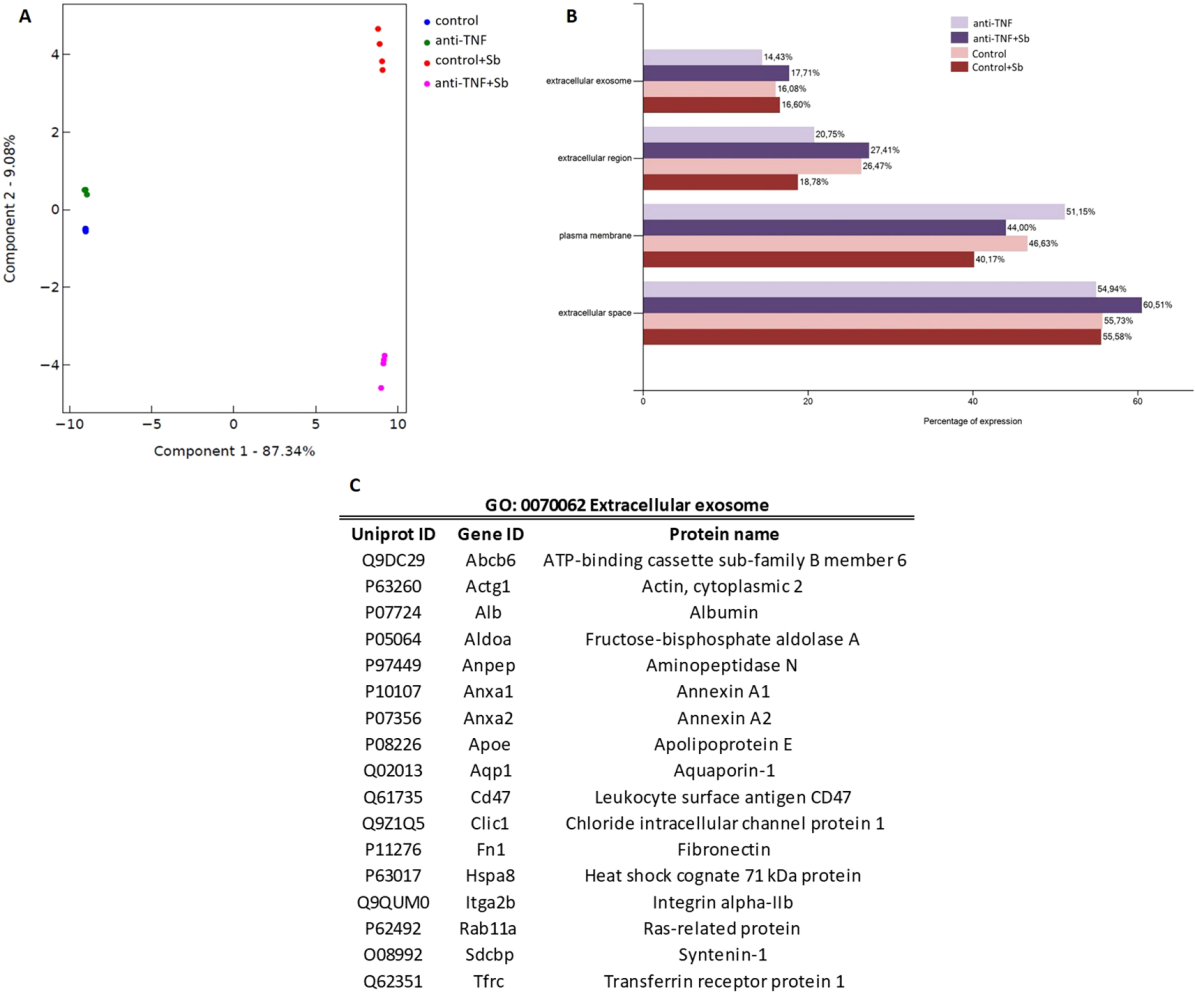
Proteomic analysis of anti-TNF and control EVs before and after antimonial treatment. **(A)** Principal component analysis (PCA) using the Flasky tool of four replicates of proteomic data obtained in each group. **(B)** Using the DAVID database, proteins identified in all samples were classified according to the Cellular Component term related to EVs and plasma. The FunRich tool was used to represent the results. **(C)** Summary of the proteins detected in all the preparations mapped to the GO term Extracellular exosome.

Next, a label-free proteomics analysis served to identify and quantify a total of 223 and 173 proteins in samples before (control and anti-TNF) and after (control+Sb and anti-TNF+Sb) antimonial treatment, respectively (**Supplementary Tables 1**, **2**). Before data analysis, we performed a principal component analysis (PCA) to ensure the distribution of samples and the reproducibility of replicates (**Figure 3A**). We observed different and distinguishable clusters between the four groups, with pre-treatment samples clustering separately from post-treatment samples, thus allowing us to compare the protein contents of EVs between samples. Further analysis to map these proteins against the *Mus musculus* genome as background was performed according to Gene Ontology (GO) terms, using the Database for Annotation, Visualization and Integrated Discovery (DAVID) and Uniprot database. An initial Cellular Component (CC) analysis was performed to classify proteins within the GO terms related to EVs and plasma such as Extracellular exosome (GO: 0070062), Extracellular region (GO: 0005576), Extracellular space (GO: 0005615), and Plasma membrane (GO: 0005886) (**Figure 3B**). The percentages of all these GO terms were similar among all samples, the term Extracellular space being the one with the greater proportion of proteins mapped (54.94% to 60.51%). 40.17 to 51.15% of these proteins were also associated with the term Plasma membrane and 18.78 to 27.41% to Extracellular region. For the GO term Extracellular exosome (14.43 to 17.71%), 17 proteins were found in all the samples (p= 1.73E-14, FDR= 8.44E-13 and p= 3.70E-16, FDR= 2.42E-14 when comparing control and anti-TNF and control+Sb and anti-TNF+Sb, respectively) (**Figure 3C**).

According to MISEV guidelines (18), an evaluation of the protein content was performed to confirm that all samples were enriched in EVs (**Supplementary Table 3**). In line with recommendations, at least one protein from each of the three major categories was present in the proteomics analysis of all samples. Identification of category 1 and 2 proteins indicates the presence of EVs by characterizing their lipid-bilayer membrane. Within category 1, we found tetraspanins CD82 and CD9 (Uniprot: P40237 and P40240), CD47 multi-pass membrane protein (Uniprot: Q61735), and many integrins, alpha and beta among others. A total of 16 proteins from category 1 were found in control and anti-TNF samples, while 15 were found in control+Sb and anti-TNF+Sb samples. In category 2, the proteins identified (21 in each of the four groups) were caveolin-1 (Uniprot: P49817), heat shock protein Hsp70 (Uniprot: P63017), together with annexins, tubulins, and actins. Finally, in category 3, which describes non-EV structures isolated with EVs, we identified albumin (Uniprot: P07724), apolipoproteins and immunoglobulins, totalling 16 in control and anti-TNF samples, and 12 in samples after Sb treatment.

### 3.2 Immunosuppression with anti-TNF leads to the reduced expression in EVs of proteins involved in biological processes associated with the response to *L. infantum* infection

**Figure 4:**
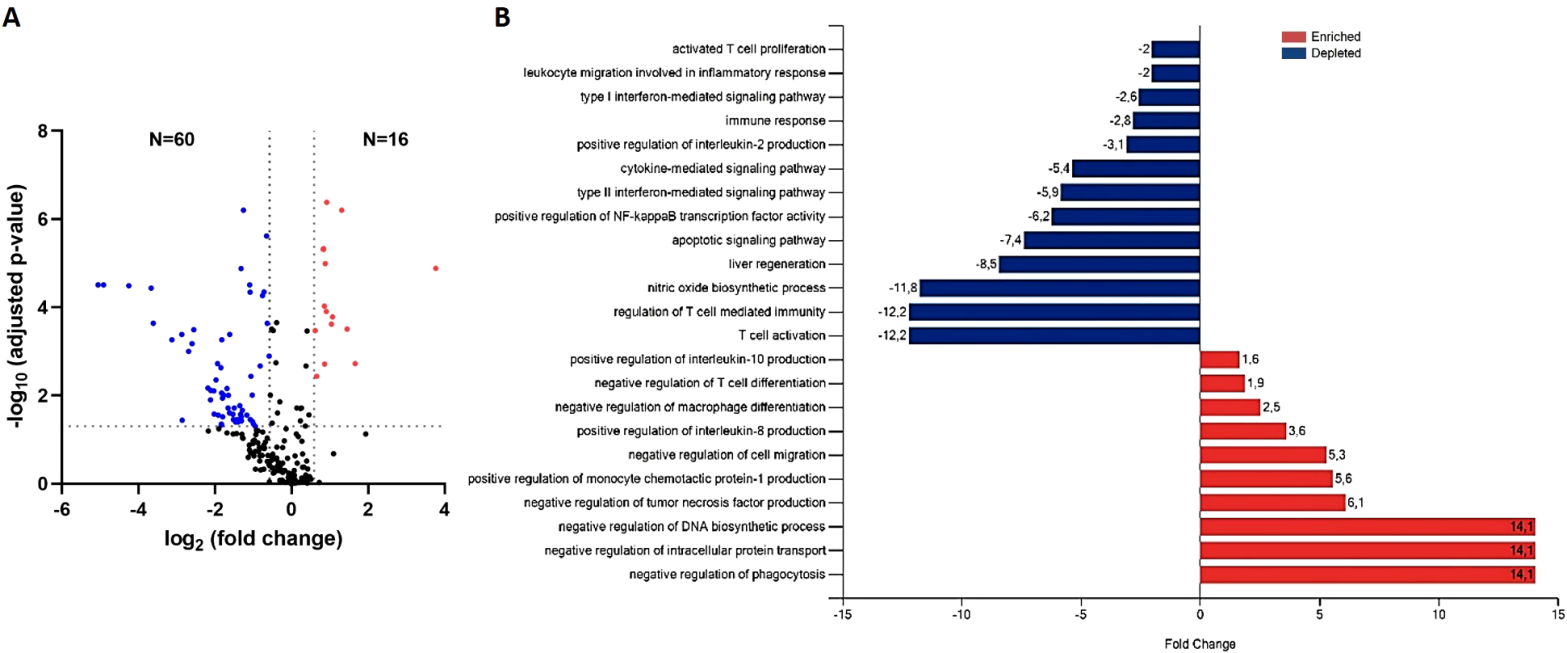
Differential expression of EV-derived proteins linked to immunosuppression by the TNF antagonist (anti-TNF vs control group). **(A)** Volcano plot of proteins found modified in the anti-TNF compared to control group. Coloured dots indicate differentially upregulated (red) or downregulated (blue) proteins in anti-TNF (FDR≤ 0.05; indicated by a horizontal dotted line). Vertical dotted lines indicate log_2_ fold changes (± 1.5) in expression. **(B)** Main biological processes altered by anti-TNF immunosuppression according to the DAVID and Uniprot databases. The FunRich tool was used for this analysis.

To explore the proteins involved in the increased severity of *L. infantum* infection under pharmacological immunosuppression with anti-TNF, as observed in the highest parasite loads in the liver of this group (7, 12), protein EV contents were compared between immunosuppressed infected and non-immunosuppressed infected mice (anti-TNF and control, respectively) (**Figure 4**; **Supplementary Table 1**). Of the 223 common proteins identified in the proteomics analysis, 76 showed a fold change (FC) ratio ≥ 1.5 or ≤ 0.67 and significant expression level differences between groups (*p* value ≤ 0.05 and FDR ≤ 0.05) (**Figure 4**). Compared to the control group (no immunosuppression treatment), anti-TNF immunosuppression led to downregulation (FC ≤ 0.67) of 60 proteins, whereas 16 were upregulated (FC ≥ 1.5) (**Figure 4A**). Next, to identify the biological processes in which these proteins were involved, a further analysis using the DAVID database was conducted to determine the GO term Biological Process (BP) (**Figure 4B**). Anti-TNF immunosuppression led to increases in many BP related to the immune system such as negative regulation of cell migration (GO:0030336), positive regulation of IL-8 production (GO:0032757), negative regulation of macrophage differentiation (GO:0045650), negative regulation of T cell differentiation (GO:0045581), and positive regulation of IL-10 production (GO:0032733). In addition, other BPs enriched under this condition were negative regulation of DNA biosynthetic process (GO:2000279), negative regulation of intracellular protein transport (GO:0090317), and negative regulation of phagocytosis (GO:0050765). In contrast, the 60 downregulated proteins in EVs in the anti-TNF group were actively involved in, at least, 10 BPs related to the immune system. Among these BPs, we identified reductions in T cell activation (GO:0042110), regulation of T cell mediated immunity (GO:0002709), liver regeneration (GO:0097421), cytokine-mediated signalling pathway (GO:0019221), type II (GO:0060333) and (GO:0060337) IFN-mediated signalling pathways, and leukocyte migration involved in the inflammatory response (GO:0002523).

Among the proteins found to be upregulated in the anti-TNF group were the apolipoproteins Apoc1 (P34928), Apoc4 (Q61268) and Apoe (P08226), which are non-EV co-isolated structures, along with the Adipoq (Q60994) protein (**Table 1**). We also identified other upregulated proteins such as Epb41 (P48193), Vcp (Q01853), Prdx2 (Q61171) or Hmgb1 (P63158). Among the 60-downregulated proteins detected in the anti-TNF samples, we identified Dpp4 (P28843), Cav1 (P49817), Vtn (P29788), Hpx (Q1X72), Ezr (P26040), Serpina1a (P07758), or Anxa6 (P14824), among others (**Table 1**).

**Table 1:** List of proteins found dysregulated in the anti-TNF group compared to the control group.

anti-TNF/control before Sb treatment					
Uniprot ID	Gene ID	Protein name	log_2_FC (anti-TNF/C)	pValue	FDR
Q60994	Adipoq	Adiponectin	3.759	5.29E-07	1.31E-05
P34928	Apoc1	Apolipoprotein C-I	1.931	3.45E-02	7.47E-02
Q61268	Apoc4	Apolipoprotein C-IV	1.657	3.49E-04	1.90E-03
P48193	Epb41	Erythrocyte membrane protein band 4.1	1.447	3.76E-05	3.11E-04
P04919	Slc4a1	Band 3 anion transport protein	1.305	8.46E-09	6.29E-07
P70290	Mpp1	Membrane protein, palmitoylated 1	1.065	1.56E-05	1.66E-04
Q02357	Ank1	Ankyrin-1	1.039	2.71E-05	2.41E-04
P02089	Hbb-b2	Haemoglobin subunit beta-2	0.915	1.87E-09	4.17E-07
Q01853	Vcp	Transitional endoplasmic reticulum ATPase	0.906	1.11E-05	1.24E-04
P08226	Apoe	Apolipoprotein E	0.876	3.64E-07	1.02E-05
Q61171	Prdx2	Peroxiredoxin-2	0.857	3.64E-04	1.93E-03
P01869	Ighg1	Ig gamma-1 chain C region	0.853	8.04E-06	9.43E-05
P01942	Hba	Haemoglobin subunit alpha	0.839	1.52E-07	4.84E-06
P54116	Stom	Stomatin	0.829	1.05E-07	4.67E-06
P02088	Hbb-b1	Haemoglobin subunit beta-1	0.818	1.40E-07	4.84E-06
P63158	Hmgb1	High mobility group protein B1	0.653	7.74E-04	3.67E-03
Q61838	A2m	Pregnancy zone protein	-4.252	2.04E-06	3.25E-05
P28843	Dpp4	Dipeptidyl peptidase 4	-3.668	2.47E-06	3.68E-05
P49817	Cav1	Caveolin-1	-3.613	2.41E-05	2.31E-04
P29788	Vtn	Vitronectin	-3.128	8.37E-05	5.47E-04
Q9QZC1	Trpc3	Short transient receptor potential channel 3	-2.87	6.13E-05	4.14E-04
P68510	Ywhah	14-3–3 protein eta	-2.858	1.32E-02	3.64E-02
Q06890	Clu	Clusterin	-2.692	1.65E-04	9.96E-04
Q91X72	Hpx	Haemopexin	-2.603	1.07E-04	6.65E-04
P07356	Anxa2	Annexin A2	-2.557	4.04E-05	3.21E-04
P22599	Serpina1b	Alpha-1-antitrypsin 1-2	-2.186	1.49E-03	6.76E-03
Q8VDD5	Myh9	Myosin-9	-2.118	3.31E-03	1.25E-02
P26040	Ezr	Ezrin	-2.117	1.77E-03	7.72E-03
P14824	Anxa6	Annexin A6	-2.031	1.82E-03	7.82E-03
Q64277	Bst1	Bone marrow stromal antigen 1	-1.976	9.52E-04	4.42E-03
Q61702	Itih1	Inter-alpha-trypsin inhibitor heavy chain H1	-1.938	3.42E-04	1.90E-03
O70165	Fcn1	Ficolin-1	-1.902	2.41E-02	5.73E-02
Q68FD5	Cltc	Clathrin heavy chain 1	-1.847	4.71E-04	2.33E-03
P19221	F2	Prothrombin	-1.835	1.89E-02	4.68E-02
P04186	Cfb	Complement factor B	-1.834	1.77E-02	4.48E-02
O55143	Atp2a2	Sarcoplasmic/endoplasmic reticulum calcium ATPase 2	-1.829	2.10E-03	8.82E-03
Q8VDN2	Atp1a1	Sodium/potassium-transporting ATPase subunit alpha-1	-1.826	8.59E-05	5.47E-04
Q8BH64	Ehd2	EH domain-containing protein 2	-1.805	3.00E-03	1.15E-02
P62983	Rps27a	Ubiquitin-ribosomal protein eS31 fusion protein	-1.803	1.05E-02	3.05E-02
P0CG49	Ubb	Polyubiquitin-B	-1.803	1.05E-02	3.05E-02
Q8R429	Atp2a1	Sarcoplasmic/endoplasmic reticulum calcium ATPase 1	-1.744	2.46E-03	9.81E-03
P10107	Anxa1	Annexin A1	-1.691	3.15E-02	7.10E-02
P21614	Gc	Vitamin D-binding protein	-1.688	1.55E-03	6.93E-03
A6X935	Itih4	Inter alpha-trypsin inhibitor, heavy chain 4	-1.661	5.52E-03	1.93E-02
Q61147	Cp	Ceruloplasmin	-1.65	2.55E-03	9.96E-03
Q61703	Itih2	Inter-alpha-trypsin inhibitor heavy chain H2	-1.622	7.68E-03	2.49E-02
P28665	Mug1	Murinoglobulin-1	-1.619	5.96E-05	4.14E-04
O55222	Ilk	Integrin-linked protein kinase	-1.531	8.47E-03	2.66E-02
Q99P58	Rab27b	Ras-related protein Rab-27B	-1.524	1.27E-02	3.53E-02
Q64518	Atp2a3	Sarcoplasmic/endoplasmic reticulum calcium ATPase 3	-1.497	5.64E-03	1.93E-02
P62874	Gnb1	Guanine nucleotide-binding protein	-1.469	1.54E-02	3.99E-02
Q9CQW9	Ifitm3	Interferon-induced transmembrane protein 3	-1.421	1.25E-02	3.53E-02
O54724	Ptrf	Caveolae-associated protein 1	-1.399	1.54E-02	3.99E-02
P59383	Lrrn4	Leucine-rich repeat neuronal protein 4	-1.356	4.68E-03	1.71E-02
O08992	Sdcbp	Syntenin-1	-1.337	8.73E-03	2.70E-02
P07758	Serpina1a	Alpha-1-antitrypsin 1-1	-1.319	5.98E-07	1.33E-05
P62259	Ywhae	14-3–3 protein epsilon	-1.314	1.04E-02	3.05E-02
P32261	Serpinc1	Antithrombin-III	-1.308	1.39E-02	3.78E-02
P08752	Gnai2	Guanine nucleotide-binding protein G(i) subunit alpha-2	-1.297	3.51E-02	7.54E-02
P01865	Igh-1a	Ig gamma-2A chain C region	-1.288	6.55E-03	2.18E-02
P62631	Eef1a2	Elongation factor 1-alpha 2	-1.279	4.36E-02	9.09E-02
P06151	Ldha	L-lactate dehydrogenase A chain	-1.271	4.55E-02	9.31E-02
Q921I1	Tf	Serotransferrin	-1.262	7.18E-09	6.29E-07
O08688	Capn5	Calpain-5	-1.172	9.36E-03	2.79E-02
P23953	Ces1c	Carboxylesterase 1C	-1.096	1.77E-06	3.10E-05
P11835	Itgb2	Integrin beta-2	-1.080	1.27E-02	3.53E-02
P13020	Gsn	Gelsolin	-1.065	7.66E-04	3.67E-03
P01902	H2-K1	H-2 class I histocompatibility antigen	-1.029	2.46E-03	9.81E-03
Q80YX1	Tnc	Tenascin	-1.024	1.48E-02	3.92E-02
P01898	H2-Q10	H-2 class I histocompatibility antigen, Q10 alpha chain	-0.994	1.79E-02	4.48E-02
P10833	Rras	Ras-related protein	-0.951	2.05E-02	4.98E-02
P01867	Igh-3	Immunoglobulin heavy constant gamma 2B	-0.933	3.65E-02	7.75E-02
P07309	Ttr	Transthyretin	-0.916	2.64E-02	6.19E-02
P39655	Alox12	Polyunsaturated fatty acid lipoxygenase	-0.877	2.72E-02	6.31E-02
P01639	Gm5571	Immunoglobulin kappa chain variable 9-120	-0.823	4.12E-04	2.14E-03
P07759	Serpina3k	Serine protease inhibitor A3K	-0.763	4.41E-06	5.46E-05
Q9D6F9	Tubb4a	Tubulin beta-4A chain	-0.758	2.97E-02	6.77E-02
P11276	Fn1	Fibronectin	-0.723	3.21E-06	4.48E-05
P07724	Alb	Albumin	-0.658	4.30E-08	2.40E-06
P97384	Anxa11	Annexin A11	-0.639	4.50E-02	9.29E-02
P01029	C4b	Complement C4-B	-0.639	2.48E-05	2.31E-04

### 3.3 Anti-TNF therapy dysregulates VL-related proteins after treatment with antimonials

**Figure 5:**
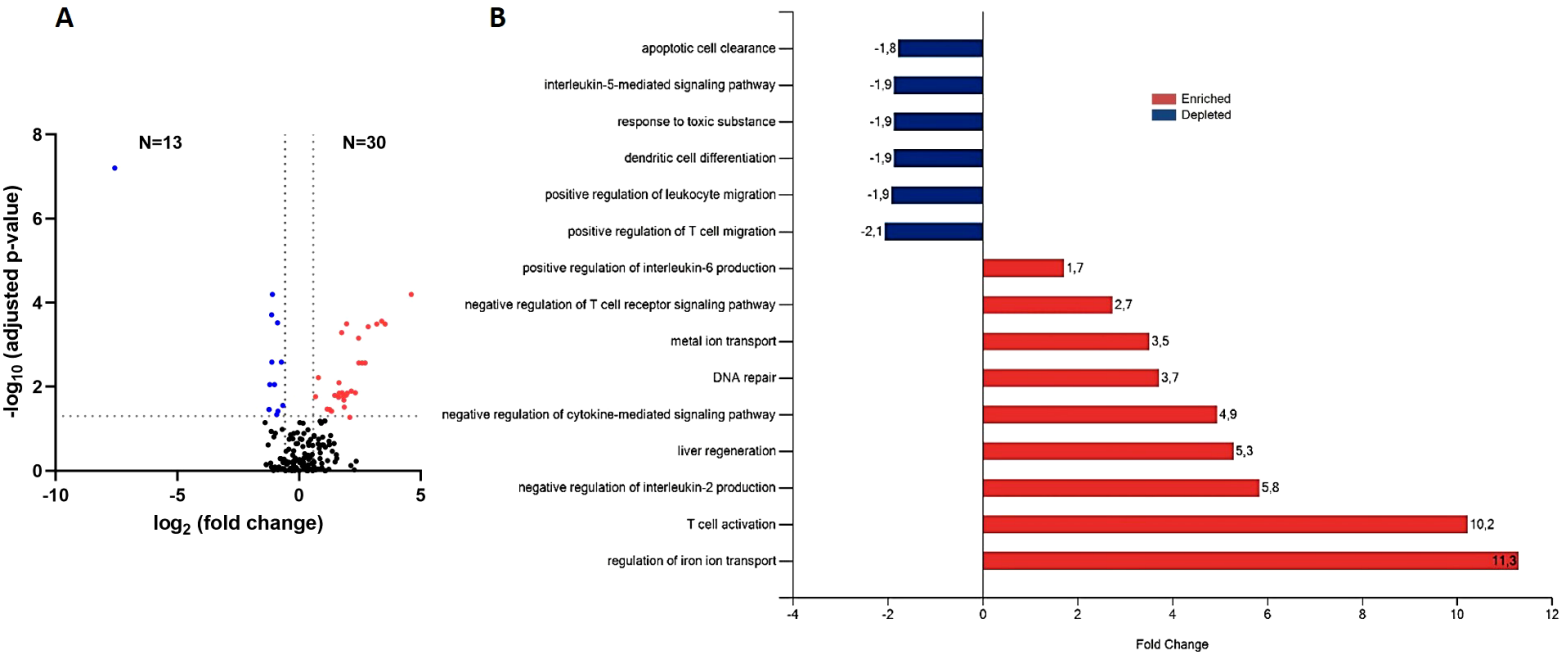
Differential expression of EV-derived proteins linked to anti-TNF immunosuppression after antimonial treatment (anti-TNF+Sb vs control+Sb group). **(A)** Volcano plot of proteins found modified in the anti-TNF+Sb compared to control+Sb group. Coloured dots indicate the differentially upregulated (red) or downregulated (blue) proteins in anti-TNF+Sb (FDR ≤ 0.05; indicated by a horizontal dotted line). Vertical dotted lines indicate log2 fold changes (± 1.5) in expression. **(B)** Main biological processes altered by anti-TNF immunosuppression after Sb treatment according to the DAVID and Uniprot databases. The FunRich tool was used for this analysis.

Our next goal was to determine whether any proteins could be responsible for a reduced response to VL treatment under this type of immunosuppression, as anti-TNF altered the immune response towards the Th2-type profile (12). To do this, we compared protein EV-contents in anti-TNF immunosuppressed-infected mice and control animals after VL treatment with pentavalent antimony (anti-TNF+Sb and control+Sb groups, respectively) (**Figure 5**). This proteomics analysis identified 173 proteins (**Supplementary Table 2**), of which 43 showed a FC ratio ≥ 1.5 or ≤ 0.67 and significantly different expression levels between the groups (*p* value ≤ 0.05 and FDR ≤ 0.05). Anti-TNF therapy led to the upregulation of 30 proteins in infected mice after antimonial treatment (**Figure 5A**). These proteins were associated with increases in the BPs regulation of iron ion transport (GO:0034756), T cell activation (GO:0042110), liver regeneration (GO:0097421), metal ion transport (GO:0030001), DNA repair (GO:0006281), positive regulation of IL-6 production (GO:0032755), and negative regulation of IL-2 production (GO:0032703) (**Figure 5B**). In contrast, only 13 proteins were downregulated in the anti-TNF+Sb group, involved in T cell migration (GO:2000406), leukocyte migration (GO:0002687), dendritic cell differentiation (GO:0097028), response to toxic substance (GO:0009636), IL-5 mediated signalling pathway (GO:0038043), and apoptotic cell clearance (GO:0043277).

The following proteins (**Table 2**) were identified among the 30 upregulated proteins in the anti-TNF+Sb samples: Tf (Q921I1), Dpp4 (P28843), Ezr (P26040), Fn1 (P11276), Cfh (P06909), Serpina3k (P07759), and Cd5l (Q9QWK4). In contrast, Ehd4 (Q9EQP2), Itgb3 (O54890), Itga2b (Q9QUMO), and Slc4a1 (P04919), were identified among the most downregulated proteins in the anti-TNF+Sb group.

**Table 2:** List of proteins found dysregulated in the anti-TNF+Sb group compared to the Control+Sb group.

anti-TNF/control after Sb treatment					
Uniprot ID	Gene ID	Protein	log_2_FC (anti-TNF/C)	pValue	FDR
P01868	Ighg1	Ig gamma-1 chain C region	4.606	1.10E-06	6.35E-05
Q921I1	Tf	Serotransferrin	3.537	1.68E-05	3.24E-04
P28843	Dpp4	Dipeptidyl peptidase 4	3.392	7.97E-06	2.76E-04
P07758	Serpina1a	Alpha-1-antitrypsin 1-1	3.191	1.62E-05	3.24E-04
P23953	Ces1c	Carboxylesterase 1C	2.836	2.16E-05	3.74E-04
P01867	Igh-3	Immunoglobulin heavy constant gamma 2B	2.709	2.65E-04	2.72E-03
P26040	Ezr	Ezrin	2.586	2.67E-04	2.72E-03
P07356	Anxa2	Annexin A2	2.452	2.36E-04	2.72E-03
P29788	Vtn	Vitronectin	2.443	4.85E-05	6.99E-04
P07759	Serpina3k	Serine protease inhibitor A3K	2.315	1.93E-03	1.39E-02
P22599	Serpina1b	Alpha-1-antitrypsin 1-2	2.146	1.63E-03	1.28E-02
Q9QWK4	Cd5l	CD5 antigen-like	1.971	1.30E-05	3.20E-04
P07724	Alb	Albumin	1.953	2.09E-03	1.42E-02
Q01853	Vcp	Transitional endoplasmic reticulum ATPase	1.932	2.48E-03	1.59E-02
P14094	Atp1b1	Sodium/potassium-transporting ATPase subunit beta-1	1.854	6.02E-03	3.06E-02
P01865	Igh-1a	Ig gamma-2A chain C region	1.838	3.85E-03	2.08E-02
Q8VDN2	Atp1a1	Sodium/potassium-transporting ATPase subunit alpha-1	1.813	2.95E-03	1.72E-02
Q8CIM7	Cyp2d26	Cytochrome P450 2D26	1.768	1.93E-03	1.39E-02
E9Q414	Apob	Apolipoprotein B-100	1.744	3.28E-05	5.15E-04
P10126	Eef1a1	Elongation factor 1-alpha 1	1.644	2.13E-03	1.42E-02
Q00623	Apoa1	Apolipoprotein A-I	1.641	8.81E-04	8.02E-03
P28665	Mug1	Murinoglobulin-1	1.625	3.19E-03	1.78E-02
P11276	Fn1	Fibronectin	1.460	2.61E-03	1.61E-02
P14824	Anxa6	Annexin A6	1.362	1.77E-02	6.64E-02
P26041	Msn	Moesin	1.334	8.94E-03	3.82E-02
Q68FD5	Cltc	Clathrin heavy chain 1	1.311	8.49E-03	3.77E-02
P13020	Gsn	Gelsolin	1.256	7.39E-03	3.50E-02
O55111	Dsg2	Desmoglein-2	1.222	2.15E-02	7.43E-02
O54724	Ptrf	Caveolae-associated protein 1	1.173	1.97E-02	7.17E-02
P06909	Cfh	Complement factor H	1.154	6.90E-03	3.41E-02
P41317	Mbl2	Mannose-binding protein C	1.086	1.99E-02	7.17E-02
Q8K0E8	Fgb	Fibrinogen beta chain	1.080	1.71E-02	6.56E-02
E9PV24	Fga	Fibrinogen alpha chain	1.058	1.66E-02	6.53E-02
P01872	Ighm	Immunoglobulin heavy constant mu	0.795	6.31E-04	6.06E-03
Q61838	A2m	Pregnancy zone protein	0.674	2.98E-03	1.72E-02
Q3UV17	Krt76	Keratin-76	-7.575	3.60E-10	6.22E-08
Q9EQP2	Ehd4	EH domain-containing protein 4	-1.235	7.68E-03	3.50E-02
Q8BTM8	Flna	Filamin-A	-1.232	7.56E-03	3.50E-02
Q8K1B8	Fermt3	Fermitin family homologue 3	-1.206	1.03E-03	8.89E-03
P02089	Hbb-b2	Haemoglobin subunit beta-2	-1.131	4.51E-06	1.95E-04
P54116	Stom	Stomatin	-1.122	2.09E-04	2.58E-03
P01942	Hba	Haemoglobin subunit alpha	-1.098	1.10E-06	6.35E-05
O54890	Itgb3	Integrin beta-3	-1.01	1.08E-03	8.89E-03
P01029	C4b	Complement C4-B	-0.914	1.12E-02	4.61E-02
P02088	Hbb-b1	Haemoglobin subunit beta-1	-0.887	1.05E-05	3.02E-04
Q9QUM0	Itga2b	Integrin alpha-IIb	-0.871	9.06E-03	3.82E-02
P04919	Slc4a1	Band 3 anion transport protein	-0.727	2.05E-04	2.58E-03
P14106	C1qb	Complement C1q subcomponent subunit B	-0.666	5.35E-03	2.80E-02

### 3.4 Analysis of dysregulated proteins in serum samples

To determine whether dysregulated proteins found in EVs could be detected in clinical samples and therefore be of use to identify situations of anti-TNF immunosuppression during *L.infantum* infection, we evaluated seven proteins (Fn, Vtn, Tf, Hpx, Cav, Dpp4 and Hmgb1) in mouse serum samples through ELISA.

**Figure 6:**
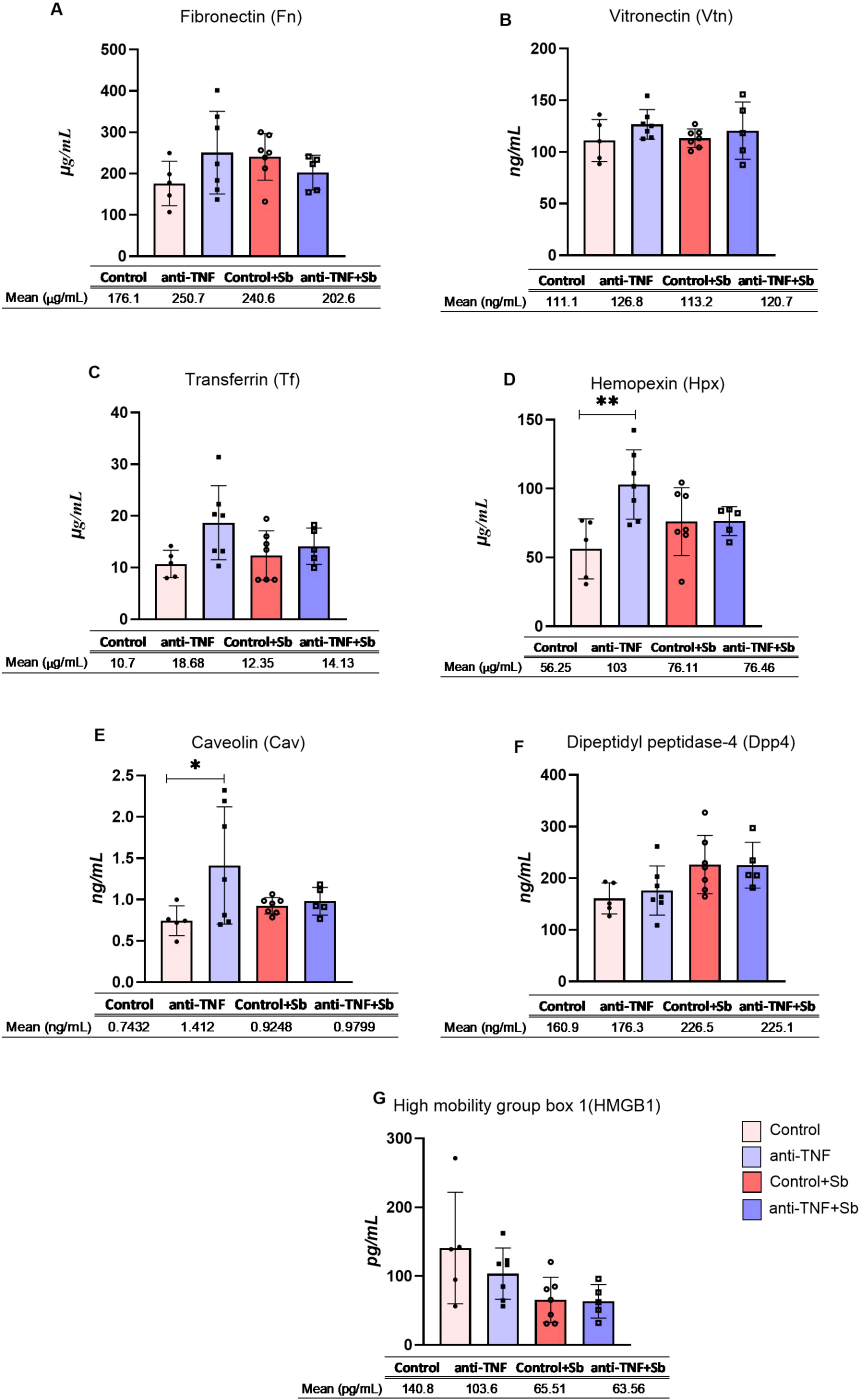
Serum concentrations of selected proteins. Serum samples were obtained after blood collection at two time points post-infection, week 6 (control and anti-TNF groups) and week 9 (control+Sb and anti-TNF+Sb groups). Seven proteins were evaluated by sandwich ELISA: **(A)** Fibronectin, **(B)** Vitronectin, **(C)** Transferrin, **(D)** Hemopexin, **(E)** Caveolin, **(F)** Dipeptidyl pptidase-4 and **(G)** High mobility group box 1. The concentration of each selected protein is shown individually. Also provided are the means and standard errors for each study group. Comparisons were made between groups sharing the same immunosuppression status (control vs. control + Sb and anti-TNF vs. anti-TNF + Sb), as well as between groups before (control vs. anti-TNF) and after (control + Sb vs. anti-TNF + Sb) VL treatment. Significant differences were assessed using an ANOVA followed by a Tukey’s *post hoc* test and are indicated as *p ≤ 0.05; **p ≤ 0.01.

Serum expression levels of Fn, Vtn and Tf (**Figures 6A–C**) showed no significant variation across the different groups. Expression levels of Hpx (103 µg/mL) and Cav (1.41 ng/mL) were significantly higher in the serum samples in the anti-TNF group compared to the control group (p= 0.0088 and p= 0.049 respectively) (**Figures 6D, E**). In addition, there is a tendency to an increase of the protein levels of Dpp4 after Sb treatment, whereas a partial reduction was observed in Hmgb1 in the post-treatment groups, with no statistical changes observed (**Figures 6F, G**). The differences in serum expression levels observed between groups did not align with the findings from the proteomic analysis of EVs.

## 4 Discussion

Quantitative proteomics analysis of EVs provides useful information on proteins associated with disease and the biological processes in which they are involved. However, while most EV studies have focused on cancer patients (29, 30), data are now starting to emerge on EV proteomic profiles in infectious diseases, such as those caused by parasites (31, 32). In the context of leishmaniasis, the few studies performed to date have mainly investigated how EVs released by *Leishmania* parasites modulate cell communication and the host immune response *in vitro* (33, 34). Although proteomics approaches have been employed in EVs recovered from canine and human VL patients (35) (Torres et al. Front Immunol, in review), these studies have not addressed the effects and complexity of this disease in conditions of immunosuppression. Such studies are essential to better understand the changes that occur during VL and its treatment in this subset of patients showing a higher risk of relapse due to anti-TNF therapy. In the present study, we compared the protein contents of plasma-derived EVs from *L. infantum*-infected mice undergoing, or not, anti-TNF therapy both before and after Sb treatment. Our ultimate goal was to identify proteins linked to disease progression and to antileishmania agent efficacy in conditions of anti-TNF immunosuppression.

Our study reveals that anti-TNF immunosuppression significantly impacts the natural progression of VL. Blocking TNF prevents granuloma formation and leads to parasite dissemination throughout the liver, causing histological lesions and hepatic necrosis (36, 37). Compared to the situation in control animals, one of the biological processes found to be negatively regulated, as reflected by the EV contents of our anti-TNF group, was liver regeneration. Accordingly, vitronectin (Vtn), a glycoprotein derived from hepatocytes and then secreted into the circulation (38), was significantly depleted in these animals. The authors of studies conducted in Vtn^-/-^ animal models have reported that deletion of the Vtn gene gives rise to delayed wound healing and tissue repair processes (39, 40). Another protein found downregulated in this anti-TNF group was haemopexin (Hpx), which is usually expressed in the liver following an inflammatory event. This protein has a high affinity for haem, which not only protects the cell from oxidative stress but also inhibits the growth of infectious agents like *Plasmodium falciparum*, thereby preventing their pathogenesis (41–43). The fact that Vtn and Hpx were depleted in the EV-contents of our anti-TNF group may explain the higher liver parasite loads found in these immunocompromised animals (9.82 x 10^4^ vs 3.71 x 10^4^ total parasites in liver in the anti-TNF vs control groups; *p*=0.0099 (12)). However, this was not observed in the serum expression levels of both proteins, as Hpx was more expressed in the anti-TNF group, whereas serum levels of Vtn remained comparable among all groups. Given that serum is not an EV-enriched matrix and may contain co-isolated proteins or degradation products, discrepancies between EV proteomic profiles and ELISA-based quantification are expected, particularly when targeting low-abundance or EV-specific proteins. Moreover, we also found that peroxiredoxin 2 (Prdx2) protein was upregulated in the EVs of the anti-TNF group compared to control animals. Peroxiredoxins (Prdx) are known to be determinants of the survival and virulence of parasites such as *Leishmania*, by protecting them from the oxidative damage caused by the host immune system (44). Other studies have shown that *P. falciparum* can import host Prdx to increase its infectivity by boosting its antioxidant defence capacity (45, 46). Addressing the question of whether *Leishmania* parasites could also sequester Prdx from the host would provide valuable insight, as this mechanism might also be involved in the increased presence of the parasite detected here in the livers of anti-TNF immunosuppressed mice.

In addition, several immune-mediated pathways were found modified in the anti-TNF immunosuppressed mice such as those of negative regulation of T cell differentiation and positive regulation of both IL-10 and IL-8 production. IL-8 and IL-10 cytokines play a critical role in VL progression as they are strongly correlated with parasite persistence (47). Enrichment in the BP IL-8 production in our anti-TNF serum-derived EV samples could be mainly related to the increased cytokine production that takes place during VL progression due to neutrophil infiltration (48). One of the proteins found upregulated in the anti-TNF group was high mobility group box 1 (Hmgb1). This protein is generally secreted in a damage-associated molecular pattern by activated macrophages and neutrophils, thus triggering a pro-inflammatory response to control infection (49, 50). However, in the context of leishmaniasis, the oxidative environment generated during intracellular infection via reactive oxygen species, changes the conformational state of the protein such that it is unable to interact with its receptors (51). This status shifts to an anti-inflammatory function, increasing the production of T regulatory cells (Tregs) and IL-10 (52). The resultant M2 phenotype leads to reduced nitric oxide production (53) and diminishes the immune system response by inhibiting the frequency of effector T cells, which contributes to parasite persistence and activation of the VL-associated pathology (54, 55) under anti-TNF immunosuppression. In addition, the downregulation of caveolin-1 (Cav1) protein further exacerbates this immune deficiency, by reducing lymphocyte activation and weakening the immune response, thereby diminishing the host’s ability to deal with *Leishmania* infection (56). This situation leads to an increased parasite burden and pathogenesis, as previously observed in our anti-TNF immunosuppressed mice at six weeks post-infection (7).

Following treatment with Sb, fibronectin (Fn) was enriched in the anti-TNF group compared to the control mice. *Leishmania* promastigotes interact with this protein to impair the activation of parasite-infected macrophages (57). Thus, Fn overexpression could lead to parasite persistence by binding to parasite receptors, promoting macrophage invasion and parasite spreading (57, 58). Another mechanism of *Leishmania* persistence is the acquisition of iron (59) by fusion of the host transferrin (Tf) receptor to the parasitophorus vacuole (60). Upregulation of this main iron-carrier protein in the post-Sb treatment anti-TNF group could play a role in parasite survival and proliferation. We also found an increase in Vtn, a protein associated with liver regeneration, in line with the partial reduction of parasite load observed after Sb treatment in immunosuppressed mice (12). Alternatively, Vtn can also bind pathogens promoting their internalization and evading a complement response via the integrin complex (61, 62). In effect, the affinity of the Vtn protein for *L. donovani* promastigotes has been reported to lead to increased circulating levels of parasites in post kala-azar dermal leishmaniasis (PKDL) patients (63, 64). Further, high levels of Vtn have been associated with the regulatory cytokine TGF-β, which plays an important role during both PKDL and VL progression, as it suppresses iNOS and IFN-γ Th1-type responses (64–66). Thus, the poorer immune response in these anti-TNF mice, together with Fn, Tf and Vtn enrichment could explain why our anti-TNF immunosuppressed mice were unable to effectively eliminate parasites despite receiving leishmanicidal treatment. Moreover, dipeptidyl peptidase-4 (Dpp4) upregulation also exacerbates the reduced capacity of anti-TNF immunosuppressed mice to eliminate the infection, as its increased presence has been observed in unresolved cases of leishmaniasis compared to levels in cured patients and controls (67). We also observed downregulation of the band 3 anion protein transport (Slc4a1) and of myosin-9 (Myh9) in our anti-TNF immunosuppressed mice after Sb treatment. These cure-related proteins have been recently described in plasma-derived EVs obtained in immunocompetent patients when compared to levels observed in patients with active VL treated with Ambisome^®^ (Torres et al. Front Immunol, in review). The impaired enrichment of these proteins after Sb treatment supports the idea of a limited response to VL treatment under conditions of anti-TNF immunosuppression and may be related to the higher risk of VL relapse seen in clinical cases.

In summary, our study provides the first integrative evidence that anti-TNF immunosuppressive therapy not only reshapes host immune responses during *Leishmania* infection but also profoundly alters the extracellular vesicle proteome, revealing a dysregulation of key biological pathways—particularly those involved in liver regeneration and iron metabolism—that may contribute to increased disease severity and reduced responsiveness to antimonial treatment. These findings underscore the need to tailor clinical management strategies for VL in individuals receiving anti-TNF therapy and highlight the potential of EV proteomics to uncover mechanistic insights and identify novel prognostic biomarkers.

## Data Availability

The mass spectrometry proteomics data have been deposited at the ProteomeXchange Consortium via the PRIDE partner repository with the dataset identifier PXD060935.
